# Microfabricated Vapor Cells with Reflective Sidewalls for Chip Scale Atomic Sensors

**DOI:** 10.3390/mi9040175

**Published:** 2018-04-11

**Authors:** Runqi Han, Zheng You, Fan Zhang, Hongbo Xue, Yong Ruan

**Affiliations:** 1 Collaborative Innovation Center for Micro/Nano Fabrication, Device and System, Tsinghua University, Beijing 100084, China; hrq13@mails.tsinghua.edu.cn (R.H.); zhang-f15@mails.tsinghua.edu.cn (F.Z.); 2 State Key Laboratory of Precision Measurement Technology and Instrument, Tsinghua University, Beijing 100084, China; 3 Department of Precision Instrument, Tsinghua University, Beijing 100084, China; 4 State Key Laboratory of Space Weather, National Space Science Center, Chinese Academy of Sciences, Beijing 100190, China; hbxue@nssc.ac.cn

**Keywords:** MEMS fabrication, rubidium vapor cells, reflectance, linear absorption contrast, coherent population trapping (CPT) spectroscopy, chip scale atomic sensors

## Abstract

We investigate the architecture of microfabricated vapor cells with reflective sidewalls for applications in chip scale atomic sensors. The optical configuration in operation is suitable for both one-beam and two-beam (pump & probe) schemes. In the miniaturized vapor cells, the laser beam is reflected twice by the aluminum reflectors on the wet etched 54.7° sidewalls to prolong the optical length significantly, thus resulting in a return reflectance that is three times that of bare silicon sidewalls. To avoid limitations faced in the fabrication process, a simpler, more universal and less constrained fabrication process of microfabricated vapor cells for chip scale atomic sensors with uncompromised performance is implemented, which also decreases the fabrication costs and procedures. Characterization measurements show that with effective sidewall reflectors, mm^3^ level volume and feasible hermeticity, the elongated miniature vapor cells demonstrate a linear absorption contrast improvement by 10 times over the conventional micro-electro-mechanical system (MEMS) vapor cells at ~50 °C in the rubidium D1 absorption spectroscopy experiments. At the operating temperature of ~90 °C for chip scale atomic sensors, a 50% linear absorption contrast enhancement is obtained with the reflective cell architecture. This leads to a potential improvement in the clock stability and magnetometer sensitivity. Besides, the coherent population trapping spectroscopy is applied to characterize the microfabricated vacuum cells with 46.3 kHz linewidth in the through cell configuration, demonstrating the effectiveness in chip scale atomic sensors.

## 1. Introduction

Atomic sensors, which are based on atomic spectroscopy and light-atom interaction, serve as precision measurement sensors in various applications [1,2]. Normally, atomic sensors are categorized as atomic clocks, magnetometers, gyroscopes, interferometers, and other types of sensors [3]. However, they can also be divided into two categories depending on whether the atoms are warm (room temperature level) or laser-cooled in vapor cells. Atomic sensors have achieved a better performance than that of traditional sensors, and thus are attracting more interest in the field. For example, the cesium frequency standards have achieved a stability of 10^−14^@1 day, while the oven-controlled crystal oscillator (OCXO) behaves worse in the long term with a stability of 10^−10^@1 day [4]. The sensitivity of the spin exchange relaxation free (SERF) atomic magnetometer has surpassed that of the superconducting quantum interference device (SQUID), which is known as the state-of-the-art most sensitive magnetometer [5]. To solve the problems of conventional atomic sensors, namely large volume, high power consumption, and high cost, micro-electro-mechanical-system (MEMS) technology has been introduced to the field of atomic sensors and leads to the fast development of chip scale atomic sensors. Those MEMS atomic sensors are featured by small volume, low power consumption, low cost, and high-level integration, and are still able to offer satisfactory performance in terms of stability and sensitivity [6,7]. Therefore, they are widely applied in fields, such as navigation, geophysics, biomedical care, etc [3]. In this paper, we focus on the applications of atomic clocks and atomic magnetometers, which are taken as representatives of chip scale atomic sensors.

In order to miniaturize the atomic clock, the principle of coherent population trapping (CPT) is more widely implemented than improving the conventional optical-microwave scheme, such as the microloop-gap resonator [8,9]. This principle only requires a simple physical architecture and promises compatibility with the MEMS technology. The vapor cells can be batch-fabricated, while all optics, including the vertical cavity surface emitting laser (VCSEL), can be integrated as small as cm^3^ to mm^3^ in volume. These also apply to atomic magnetometers, operating in the CPT structure, traditional optical pumping regimes, or even SERF technology, leading to the investigation of chip scale atomic magnetometers [10,11]. In particular, the optical architecture for atomic magnetometers is composed of one pump beam to generate coherence and one probe beam to detect the optical rotation signal. The conventional MEMS vapor cell structure consists of stackable optics and a glass-silicon-glass three-layer sandwich, which enables through-pass beam propagation [12]. The optical length depends on the silicon wafer thickness, mostly being less than 2 mm due to the limitation of MEMS fabrication technology. Since conventional vapor cells typically have an optical length on centimeter level, microfabricated vapor cells lead to a lower signal-to-noise ratio, resulting in less competitive performance [13].

Several constructive approaches have been implemented to overcome this shortcoming. In 2004, an effective architecture of folded optics with a reflective mirror was presented whose optical length equaled as twice the thickness of silicon body, while the laser and photodetector were placed on the same side of the cell [14]. In 2007, the diverging VCSEL beam was utilized as the pump beam as well as the probe beam simultaneously to realize differential detection and cancel the common-mode noise [15]. Honeywell proposed an integrated three-layer stack configuration with two mirrors in the middle wafer [16]. In 2008, a micro-structured dual-focus optics based on micro-Fresnel lens was incorporated into a dual-pass reflective configuration [17]. In 2009, Bragg reflectors, composed of layers of amorphous silicon, silicon dioxide (SiO_2_), and silicon nitride (Si_3_N_4_) were integrated, which improved the reflectance of angled sidewalls by nearly three times (as compared to that of uncoated silicon), resulting in an eight-times increase of the return reflection efficiency [18]. The variation in the dielectric thin film thickness due to the arrival angles of plasma enhanced chemical vapor deposition (PECVD) was optimized by the deposition rate [19,20,21]. Sandia National Lab reported a tunable miniature SERF atomic magnetometer with gratings and movable mirrors in the probe light path [22]. In 2011, a multi-pass cell was fabricated with a deterministic number of light passes to obtain a large optical rotation angle in excess of 100 rad [23]. In 2012, a fabrication method to realize an optical length of ~5 mm was proposed, which included four-step anodic bonding between silicon wafers and thick glass wafers [24]. In 2013, Honeywell applied for patents on a novel atomic sensor physics package. It contained a well-defined chamber with an arbitrary shape containing alkali metal with transmissive surface for incident and exiting light, as well as reflective mirrors to prolong optical path [25]. In 2013, a new vapor cell architecture with sacrificial micro-channels and glass cubes was proposed, which was further sealed by glass-frit reflow [26,27]. In 2015, the microfabricated cells with two possible optical paths (transmission and double reflection mode) were fabricated and a relative frequency stability of 1 × 10^−11^@100 s was estimated from the CPT signal of 2–3 kHz linewidth and 1500 signal-to-noise ratio in the transmission mode [28]. The research group in Franche-Comté Électronique Mécanique Thermique et Optique - Sciences et Technologies (FEMTO-ST) Institute designed and microfabricated vapor cells with diffraction gratings and a reflective optical structure that was based on 54.7° angled sidewalls coated with aluminum inside the vapor cavity [29].

This paper demonstrates microfabricated vapor cells with aluminum reflectors on wet etched sidewalls operating in the integrated optical configuration. The cell structure and optimized fabrication process are presented in details to strike a balance between batch fabrication the simplicity and device performance. Characterization and measurement of microfabricated vapor cells have verified their improved performance in terms of reflection efficiency, linear absorption contrast, CPT contrast, etc. Such vapor cells, with low fabrication costs and improved performance, are promising in chip scale atomic sensor applications.

## 2. Vapor Cell Architecture

The vapor cell is the core component of atomic sensors. The alkali metal is so active that it is difficult to transport pure alkali metal into the cell directly, let alone other microfabrication limitations. In order to avoid the disturbance of impurities on optical propagation, dual cavities are developed to separate the alkali metal dispenser and the light path. One cavity is used to store the alkali metal dispenser or compounds and the other cavity is used to allow for optical access to the atoms. The channels between the two cavities are narrow enough to prevent the diffusion of impurity particles.

In order to obtain relatively large beam diameter to increase the number of interacting atoms, we implement through-hole cavities instead of blind-hole cavities. There are four approaches to fabricate the desired dual cavities in a silicon substrate. (1) Laser drilling is used to fabricate tiny holes with diameters of ~0.1 mm. Even though laser drilling provides straight deep micro-holes, the heat-induced structure change, as well as residual stresses, result in bad sidewall roughness and edge collapse, which may have a bad influence on the silicon-glass anodic bonding surface [30,31]; (2) Ultrasonic drilling has relatively low positioning accuracy and fabricates smooth surfaces but quite rough sidewalls; (3) Sandblasting drilling forms rough sidewalls with an angle of 70° with respect to the substrate as well as the edge collapse; (4) Anisotropic wet etching of <100> silicon leads to sidewalls of 54.7° from the principal surface, promising extended applications. This angle is close to the ideal 45° reflection angle and the smoothness of sidewalls and surfaces maintains at an atomic-scale level, which is appropriate for reflectors. Besides, the wet etching process is compatible with MEMS fabrication and integration, which is one of the major concerns for vapor cell design. As such, the wet etching of silicon is selected as the main approach in this experiment. 

**Figure 1:**
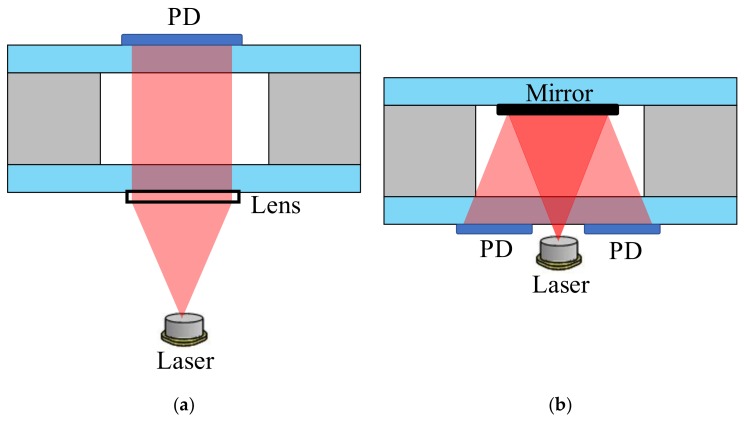
Optical configurations of (**a**) single light passing through the cell cavity and (**b**) round-trip light pass with folded optics.

The conventional optical configurations are straightforward, either directly passing through the cell cavity (single light) or going back and forth with folded optics, such as mirrors and photo detectors (round-trip light), as shown in Figure 1. The optical length is limited by the thickness of the silicon substrate, which results in a relatively low signal-to-noise ratio as compared to the conventional glass-blown vapor cells, and thus worsens the performance of chip scale atomic sensors, such as clock stability and magnetometer sensitivity. Besides, in order to increase the detected magnetic resonance amplitude, the conventional two-beam architecture in atomic magnetometry has the pump beam perpendicular to the probe beam [32]. Due to the opaque silicon substrate, as well as the limited cavity size, the microfabricated vapor cell has to be inclined with a certain angle to allow for the access of both pump and probe beams. This leads to more limitations on the beam diameter, lower MEMS integration level, and undesired incident angle of the probe beam [33]. However, if we transform the optical length from the thickness/depth to the width/length of the vapor cell cavity, the wafer thickness limitations will be avoided, thus leading to significant improvements of the optical length by orders of magnitude. Moreover, the unwanted probe incident angle, as well as beam diameter constraints, can be avoided as well. Besides, with potential high-level integration, such microfabricated vapor cells combined with optics can operate as either the one-beam chip scale atomic clock or the two-beam atomic magnetometer. 

**Figure 2:**
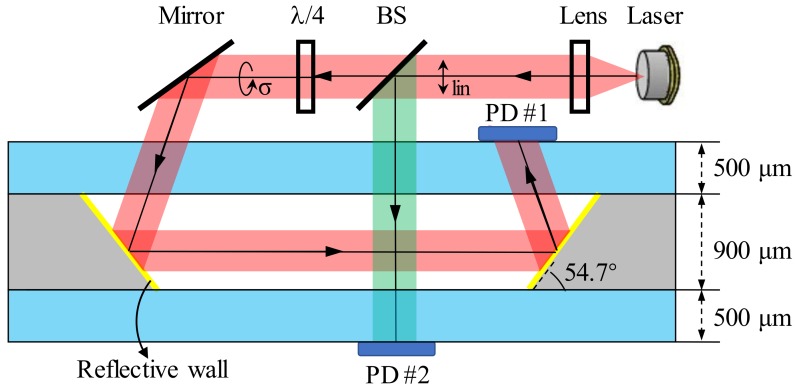
Integration architecture schematic for chip scale atomic sensors with redirected laser beams in microfabricated vapor cells. The red light represents the pump beam and the green light is the probe beam. BS: Beam splitter.

In this study, we utilize the 54.7° silicon sidewalls that were fabricated by wet etching to realize the aforementioned configuration. The vapor cell architecture is depicted in Figure 2. The silicon wafer is 900 μm thick and the glass substrate is 500 μm thick. The size or width in the opening direction of the cavity is not a fixed value, but is determined by the layout design. The light that is illuminated from the laser is split into two beams. One circularly polarized beam serves as the pump beam and it is redirected by the mirror, and reflective walls of the vapor cell subsequently. It finally gets absorbed by PD #1, which is compatible for both atomic clocks and atomic magnetometers. The photodiode and the laser are arranged on the same side. The other linearly polarized beam is the probe light and the signal is detected via PD #2. This optical path can be simplified or omitted in order to fit for the CPT atomic clock structure with only one beam. The vapor cells have the potential to be integrated with microfabricated optics or to be packaged with other optical components in a sustaining structure [34], while maintaining the flexibility and applicability. 

Though the bare silicon sidewalls that are prepared by wet etching are smooth, their reflectance is too low as reflectors. The reflectance *R* of unpolarized light at normal incidence is calculated as:

(1) $$R\;=\;\frac{{\left(n-1\right)}^{2}+k^{2}}{{\left(n+1\right)}^{2}+k^{2}}$$

where *n* is refractive index and *k* is the extinction coefficient. For silicon at rubidium D1 line of 795 nm wavelength, *n* = 3.7, *k* = 0.0068 and *R* = 0.33 [35]. This implies the silicon reflector loses at least 67% optical power after one reflection and a minimum 89% of original incident power after two times of reflections. When considering the MEMS batch fabrication and the material cost, aluminum is compatible and widely used for mirrors [36]. For aluminum at 795 nm, *n* = 2.8, *k* = 8.4, and *R* = 0.87. This indicates that the aluminum reflector that is deposited on the silicon sidewall theoretically conserves 87% optical power after one reflection and 76% of original incident power after two times of reflections. While considering the low costs to deposit aluminum using simple standard MEMS technology, our proposed device design and fabrication process are suitable for batch fabrication and actual applications. The reflectance of the aluminum film is not strongly dependent on the light wavelength, incident angle, and film thickness, resulting in great versatility of the device. Aluminum reflectors may cause magnetic thermal noise [33], but such noise is an order of magnitude lower than the chip scale atomic magnetometer sensitivity and the operating magnetic field (μT level) of chip scale atomic clocks. Therefore, aluminum is an ideal reflector material and the advantage of long optical length in the MEMS vapor cells can be fully taken to realize a high signal-to-noise ratio, resulting in an optimized stability and sensitivity. 

## 3. Vapor Cell Fabrication

**Figure 3:**
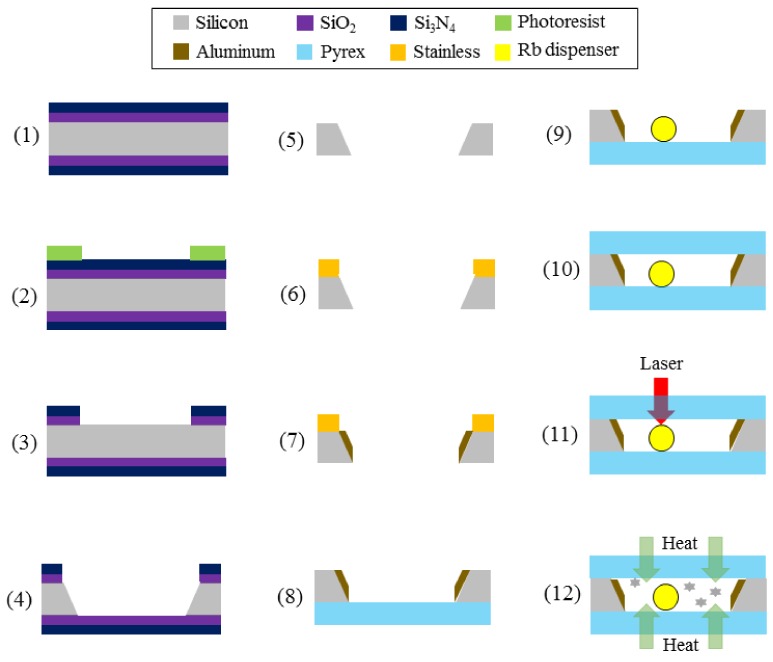
Bulk silicon microfabrication and alkali metal operation processes of miniature vapor cells with reflective aluminum sidewalls.

The vapor cell is microfabricated based on bulk silicon micromachining and alkali metal manipulation. It includes five procedures, as depicted in Figure 3: wet etching a silicon wafer for 54.7° sidewalls, depositing aluminum reflectors on the sidewalls, alkali metal transportation, vapor cell sealing with anodic bonding, and alkali metal activation. In this experiment, natural abundance rubidium is encapsulated in the vapor cell.

We start with wafer preparation and standard cleaning: a 6-inch-diameter double-side-polished *N*-type <100> Czochralski-silicon wafer with 900 μm thickness, 1–15 Ω·cm resistivity. In Step (1), a 5000 Å SiO_2_ layer and a 20,000 Å Si_3_N_4_ layer are deposited successively on both sides of the silicon substrate via PECVD by SPTS Delta LPX (SPTS Technologies Ltd., Newport, UK). In Step (2), the geometric shape of the vapor cell is patterned with 1.6 μm thick photoresist AZ5214 (MicroChemicals GmbH, Ulm, Germany) via photolithography. The film mask is aligned to the wafer top surface. In Step (3), the masks of Si_3_N_4_ and SiO_2_ layers for wet etching are etched via inductively coupled plasma (ICP) to the surface of silicon substrate, according to the photoresist pattern. In Step (4), based on the Modutek KOH (San Jose, CA, USA) wet etching platform, the wafer is immersed in the 24% potassium hydroxide (KOH) solution at 90 °C for over 9 h at an etching speed of ~1.6 μm/min to form the through hole with 54.7° sidewalls along <111> crystalline direction. The width in the opening direction of the etched cavity results from the lithography layout design. Then, in Step (5), the double-side Si_3_N_4_ and SiO_2_ mask layers are removed using buffer oxide etching (BOE) solution. 

In Step (6), a 60 μm thick SUS304 stainless mask is manufactured and is stamped onto the wafer top surface to protect the bonding contact area during aluminum deposition. The mask is aligned and then clamped by EVG620 (EV Group, St. Florian am Inn, Austria). Since the projection length of sidewalls on the horizontal direction is only 637 μm, special alignment masks are designed. The fabrication error of the stainless mask is ~20 μm, the lithographic error is ~20 μm, the KOH etching error is ~30 μm, and the alignment error is ~30 μm. In order to prevent aluminum from climbing along the sidewall, the mask is designed to cover the silicon boundary area with a 200 μm shorter margin. This ensures the aluminum reflector area to fit for a maximum beam diameter of 750 μm. 

In Step (7), 2000 Å aluminum is deposited via electron beam evaporation by Denton Integrity-26 (Denton Vacuum, Moorestown, NJ, USA). The stainless mask is then removed and a 6 inch diameter Pyrex 7740 glass substrate (Valley Design, Shirley, MA, USA) of 500 μm thickness is prepared. In Step (8), the preformed silicon wafer is anodically bonded with glass by EVG510. In Step (9), a rubidium dispenser from SAES (Florence, Italy) is placed on the concave [37]. There are four approaches to transport alkali metal into the cell. The conventional method is using BaN_6_ and RbCl to release the rubidium substance by chemical reaction at high temperature, but the compound is poisonous and a reverse reaction exists [38]. The improved method is the in-situ ultraviolet decomposition of RbN_3_, while the buffer gas pressure is difficult to be precisely controlled [39,40,41,42]. Another strategy is to use paraffin to fully cover rubidium in the glove box with MEMS molds and to transport the paraffin-rubidium package to the cell directly, regardless of atmosphere [43]. However, the melting point of paraffin requires low temperature anodic bonding. The last approach is utilizing the cylindric rubidium pill of 1 mm diameter and 0.6 mm height. It contains 33% rubidium source (0.825 mg), 56% zirconium, and 11% aluminum, which is stable in the ambient atmosphere and can only be activated to release rubidium above ~600 °C. This method is convenient and is compatible with MEMS batch fabrication, and thus it is used in the fabrication.

**Figure 4:**
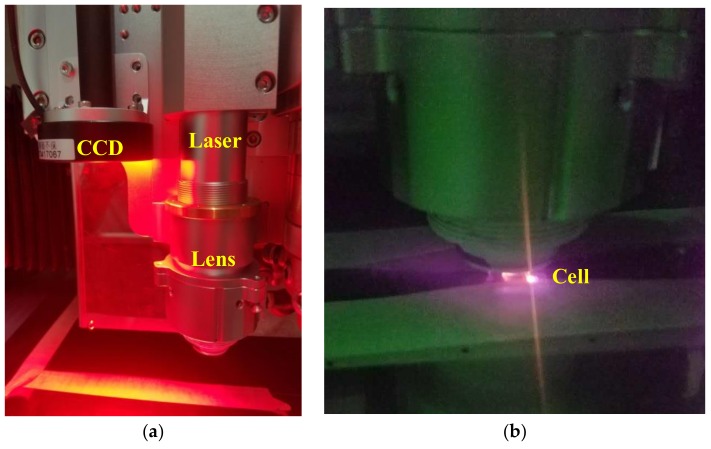
(**a**) Photo of rubidium dispenser activation setup with a laser, a lens and a charge coupled device (CCD); (**b**) Zoom-in photo of rubidium dispenser activation process.

In Step (10), the silicon-glass preform is sealed with another 500 μm thick Pyrex 7740 glass substrate by EVG510. The anodic bonding process is performed in vacuum condition (10^−3^ Pa) at 800 V voltage, 600 N force, and 355 °C temperature without buffer gas. As for anti-relaxation wall coating, such as octadecyltrichlorsilane (OTS), a low bonding temperature ~140 °C [44,45] or even lower is required to preserve the elastic wall collision property below the degradation temperature of wall coating material [46]. In Step (11), the rubidium dispenser is activated by laser heating. The activation setup is presented in Figure 4a. A 1064 nm continuous-wave fiber laser from IPG (New York, NY, USA) operates as the light source and the lens focuses the beam. A charge coupled device (CCD) of 5 million pixels assists cell position adjustment. This setup guarantees precise cell positioning (±10 μm), abundant heating power (up to 200 W), and controllable heating time. The dispenser is heated at 6 W power for 10 min to reach ~600 °C, during which it flashes, as shown in Figure 4b. In Step (12), hot air flow is used to heat the cell and to fill the whole cavity with rubidium.

## 4. Results and Discussion

### 4.1. Vapor Cell Geometry

**Figure 5:**
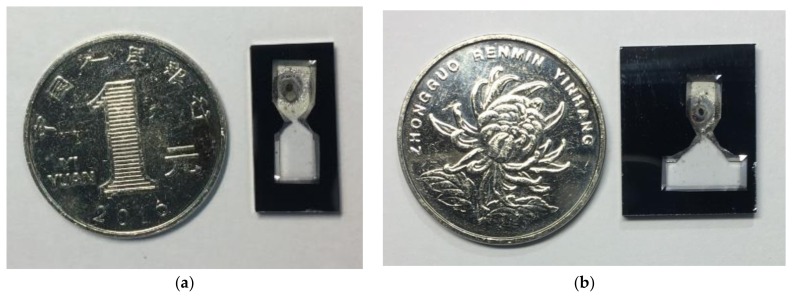
Cell photo of (**a**) 5 mm and (**b**) 10 mm long reflective optical length compared with 1 RMB coin.

56 dual-cavity cells were microfabricated in a 6-inch wafer in different sizes. The length of the cavity for redirected light pass is varied from 5 to 10 mm. The cavity volume is ~50 mm^3^. Due to the convex corner etching in anisotropic etching process, the distortion of cavity channels is evident and the convex corner compensation assists in fabricating the square corner [47]. The cells were diced and two examples are presented in Figure 5. Due to the high temperature laser heating, the rubidium dispenser is cracked and the metallic luster is visible. Further investigation on the optimization of laser heating parameters will be addressed in our future work, which would assist to relieve the damage on the dispenser’s appearance.

**Figure 6:**
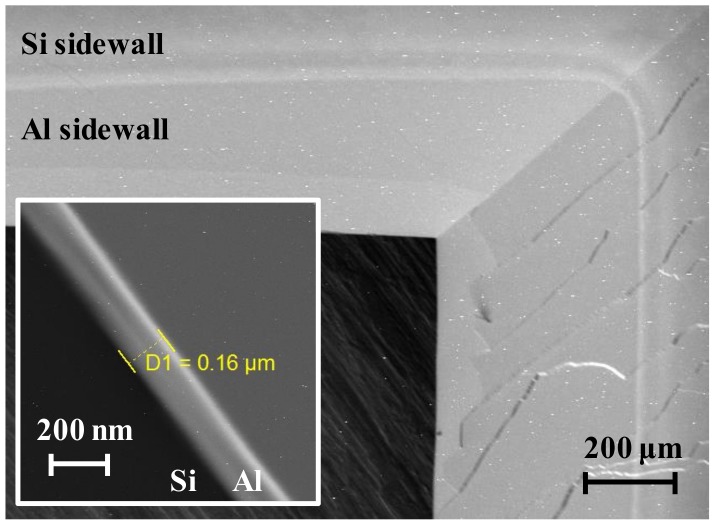
Scanning electron microscope (SEM) micrographs of sidewalls viewed from the top surface and cross section (inset).

The cell was then split and observed by a MIRA3 scanning electron microscope (SEM) (Tescan, Brno, Czech Republic). Views from both the top surface and the cross section are shown in Figure 6. The boundary of silicon and aluminum on the sidewall is clearly seen and it verifies the effectiveness of the stainless hard mask during aluminum deposition that covers the silicon boundary area with a 200 μm shorter margin. The fringes on the non-reflective sidewalls are caused by crystal competition during wet etching [48]. On account of the arrival angle reduction during electron beam evaporation, the thickness of deposited aluminum is ~1600 Å at the lower part of sidewalls. The topography of the sidewalls is measured by SEM, and the maximum fluctuation value is on the order of tens of nanometers, indicating the applicability to serve as reflectors since 10 nm is the standard roughness of perfect mirrors. The smooth angled sidewalls also contribute to the reflectance.

### 4.2. Vapor Cell Hermeticity

To provide a stable atmosphere for light-atom interaction, it is necessary for the vapor cell to maintain a steady pressure, which influences the cell lifetime and also contributes to minimizing the clock frequency shift that is caused by buffer gas pressure and temperature shift in the vapor cell [49]. In order to avoid the reverse reaction of conventional rubidium compounds, the approach of dispenser pills is applied. There are several hermeticity measurement approaches [50,51], and in our work aging tests were implemented, being mainly based on the leak rate measurement. 

The evolved cell pressure *P* with time *t* can be derived from the internal and external pressures, as well as cell cavity volume *V*. The proportional relationship is given as [52]:

(2) $$\frac{dP}{dt}\;=\;\frac{L}{P_{norm}V}\left(P_{ext}-P\right)$$

where *L* is the leak rate, *P_norm_* is the normalized pressure of 1 × 10^5^ Pa and *P_ext_* is the external pressure of 1 atm. By solving Equation (2), the pressure can be expressed as:

(3) $$P\left(t\right)\;=\;P_{ext}\left(1-e^{\frac{-Lt}{VP_{norm}}}\right)$$

where the initial pressure in the beginning is 10^−3^ Pa depending on EVG510. Due to the order of magnitudes difference between internal and external pressures, approximation is made to calculate the leak rate *L* based on the gas flow model [53]:

(4) $$L\;=\;V\frac{\Delta p}{\Delta t}$$

The leak rate was measured according to the standard helium leak test. A L300i Dry helium mass spectrometer leakage detector from Leybold Phoenix (Leybold GmbH, Cologne, Germany) was used to measure the hermeticity in terms of MIL-STD-883E regulation [54]. The leak rate over a three-month period maintained at (1.5 ± 0.2) × 10^−8^ Pa·m^3^/s for the whole diced vapor cells shown in Figure 5a. Before the anodic bonding process, the chamber of EVG510 was evacuated to 10^−3^ Pa. Based on Equation (4) and the experimental results, the estimated cell lifetime is at least three months. Due to the helium permeation of glass substrate, the measured leak rate is much larger than the actual air or oxygen leak rate with atmosphere. There are several approaches to overcome this problem, such as applying aluminosilicate glass instead of borosilicate glass [55], using gallium phosphide and Pyrex as sealing materials for anodic bonding [56], and choosing an alternative gas to implement leak test, while the reported lifetime for microfabricated vapor cells with buffer gas can be over nine months [57,58]. As such, when considering the slowing-down process, the actual lifetime could be much longer than the calculated value. 

According to the chemical equations of rubidium oxidation process and ideal gas state equation of rubidium saturation pressure, the maximum allowable air pressure is roughly estimated to be 0.25 Pa at room temperature 25 °C. The absorption spectroscopy in the following also demonstrates the hermiticity; otherwise, the spectroscopy would turn broader or even flat. Techniques to improve the hermeticity are under investigation and will be addressed in the future work.

### 4.3. Through Cell Absorption

**Figure 7:**
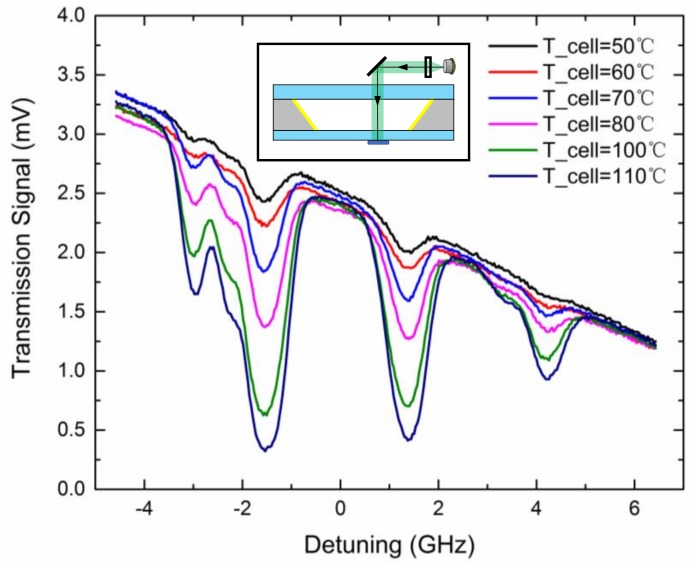
Through-cell absorption spectroscopy of rubidium D1 transition at different temperatures and the through-cell optical configuration (inset).

To characterize the atomic properties of microfabricated vapor cells, experiments of through-cell absorption spectroscopy were carried out. A 795 nm distributed feedback (DFB) laser from Eagleyard Photonics (Berlin, Germany) operating at 45.8 °C and 59 mA was modulated with a 6.4 V, 10.5 Hz ramp wave to detune as far as 6 GHz. The incident laser power was 8.5 μW and the beam diameter was 700 μm. The beam passed through the entire cell from the top surface to the bottom surface, and the transmission signal was detected by a PDA36A photodiode from Thorlabs (Newton, NJ, USA). The cell was heated with hot air flow and the temperature was measured by a FLIR-T260 thermal infrared imager (Wilsonville, OR, USA). The rubidium D1 absorption spectroscopy was recorded at different temperatures as shown in Figure 7. Due to the natural abundance of rubidium and absence of buffer gas, with an increased detuning frequency, absorption peaks of atomic transitions, namely = 2→F’, ^85^Rb F = 3→F’, ^85^Rb F = 2→F’, and ^87^Rb F = 1→F’ were clearly observed in sequence without buffer gas pressure broadening. 

As light propagates through the cell, the transmitted light intensity *I* is expressed as [59]:

(5) $$I\;=\;I_{0}e^{-OD}$$

where *I*_0_ is the initial incident light intensity. *OD* is the optical depth given as:

(6) OD = nσ(ν)l

where *n* is the atomic density, *l* is the optical length, and *σ*(*υ*) is the photon absorption cross section, which is determined by atomic frequency response on the frequency *υ*. 

Due to the limitations of silicon wafer thickness, here *l* = 0.9 mm. At room temperature, the vapor is optically thin and no absorption peaks are visible. Only heated to a relative high temperature (at least 50 °C), the vapor is not so optically thin and the amplitude of atomic transitions is measurable. This results in great power consumption and deteriorates the anti-relaxation wall coating effects. Thus, effective approaches to enhance the linear absorption contrast are in great demand.

### 4.4. Single Reflection

To characterize the reflectance of aluminum reflectors, the single reflection was measured. The same DFB laser tuned at rubidium D1 line in Section 4.3 was applied. A microfabricated vapor cell was split and mounted on a rotation stage to adjust the incident angle. The reflection of circularly polarized incident light was measured with a photodiode. The reflectance of the bare silicon 54.7° sidewall was also compared to demonstrate its efficiency. 

**Figure 8:**
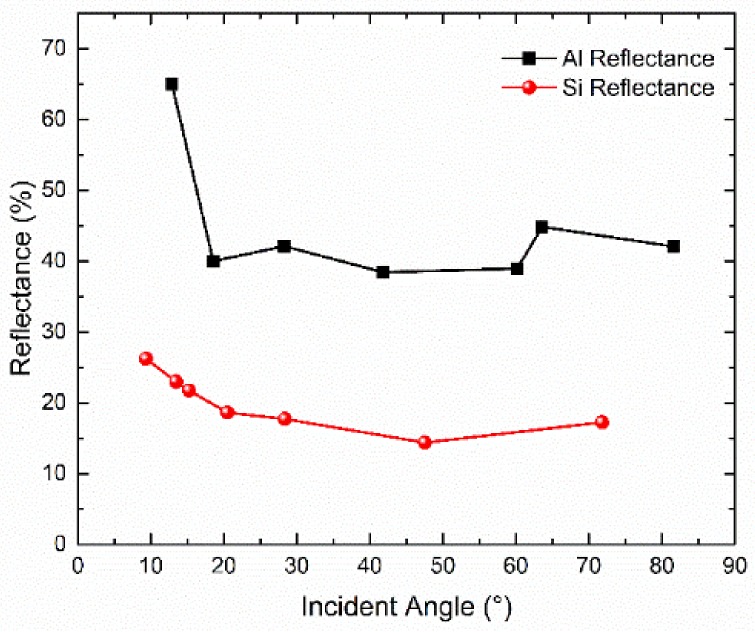
Reflectance of microfabricated silicon and aluminum reflectors at 795 nm.

Figure 8 presents the sidewall reflectance of aluminum and silicon. Due to the position accuracy and the aluminum deposition error, as well as rubidium condensation on the window, there is noticeable fluctuation in the reflectance and the experimental reflectance is smaller than the theoretical values. In spite of this, the reflectance of aluminum reflectors is twice that of silicon reflectors, which proves remarkable improvement. Besides, the state of polarization was also measured by a PAX5710IR1-T polarimeter from Thorlabs. For aluminum, the ellipticity of circular polarization is degraded to 1.3 at 35° incident angle and the polarization state changes within an acceptable range [60].

### 4.5. Return Reflection

To evaluate the performance of the microfabricated vapor cells with reflective aluminum sidewalls in the chip scale atomic sensors, we utilized optics that were similar to those in Section 4.3 and measured the return reflection of the reflectors. A vapor cell with 5 mm reflective optical length (*l* = 5 mm) was tested. The unmodulated circularly polarized 795 nm DFB laser beam was reflected by one sidewall and was redirected by the pared sidewall back to the emission plane. The incident light power was 67 μW and the beam diameter was 700 μm. The incident angle was carefully adjusted to ~35°. The measured return reflectance of aluminum reflectors was 26%, which was nearly three times as much as the ideal silicon return reflectance of 9%. The 4% reflective loss every time light passes through glass as well as alkali metal condensation on the window accounts for the difference between the experimental and the theoretical return reflectance results. The ellipticity of return reflection was degraded to 1.5 and it remained stable with less than 20% variation range. The results demonstrate the efficiency and effectiveness of microfabricated aluminum reflectors.

**Figure 9:**
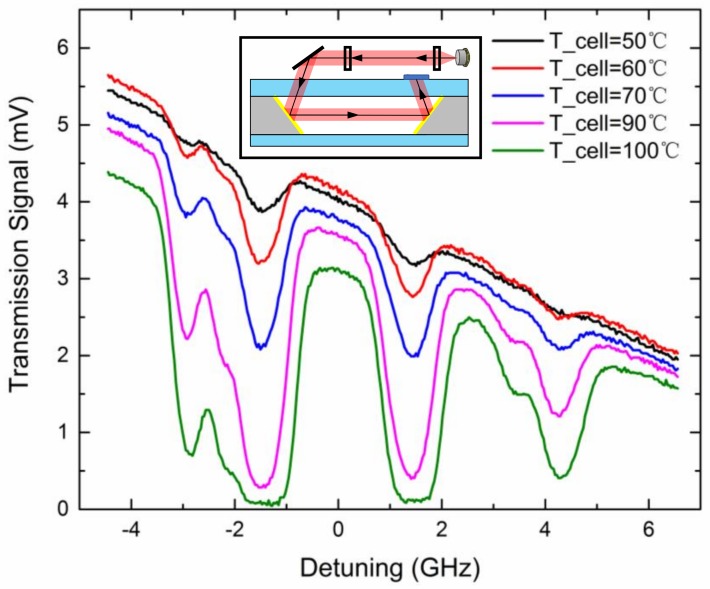
Rubidium D1 line absorption spectroscopy with 5 mm long return reflection at different temperatures and the return reflection optical configuration (inset).

Then, we modulated the laser and heated the cell in the same way, as depicted in Section 4.3, to measure the absorption spectroscopy. The results are presented in Figure 9. Similar to the through-cell absorption, four peaks correspond to four atomic transitions of natural abundance rubidium and their amplitudes increase dramatically with a temperature rise. When compared with the through-cell absorption, the *l* = 5 mm cell leads to larger peak amplitudes at each temperature. At ~90 °C, the rubidium vapor was close to the content of optically thick where the incident light fully interacted with atoms and got absorbed. The strength of Rubidium-87 F = 2→F’ transition was also strong enough to be clearly distinguished. To compare the effects of different reflective optical length, *l* = 10 mm vapor cells were also tested by the absorption spectroscopy. 

To evaluate the improvement of the reflective optical length, we extract characteristic parameters from the absorption spectroscopy. The linear absorption contrast is calculated as the absorption signal amplitude (height) divided by absorption signal background [61]. A larger linear absorption contrast results from an increased optical depth (OD), which means that more atoms are interacting with photons within a wide frequency band. During the frequency scan in the absorption spectroscopy, the particular transition frequencies for CPT phenomenon are also included. In that case, under a certain mode or route of optical pumping manipulation to establish the three-energy-level Λ structure, more atoms will be put into the coherent dark states, and thus the amplitude of transmitted CPT peaks will increase based on Equation (5). Assuming that the signal background is unchanged, this leads to a higher CPT signal-to-noise ratio and contributes to a better atomic clock frequency stability and atomic sensitivity.

**Figure 10:**
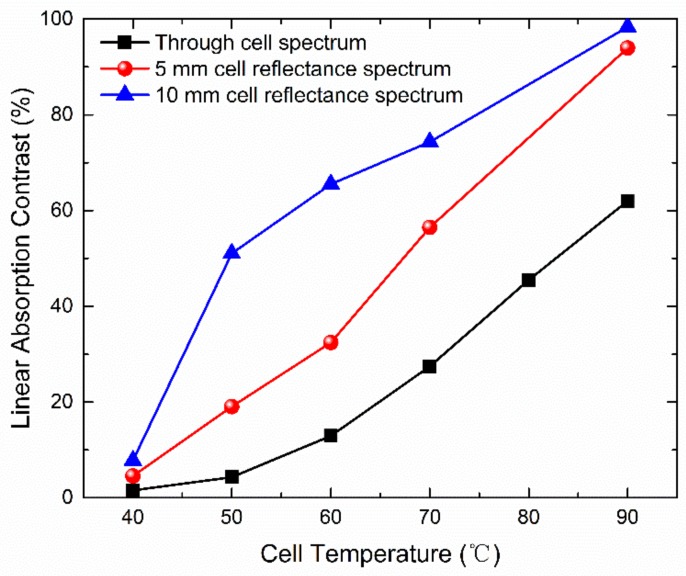
Linear absorption contrast comparison of vapor cells with different optical lengths at different temperatures.

The linear absorption contrast comparison of through cell (*l* = 0.9 mm), *l* = 5 mm and *l* = 10 mm return reflection configurations is presented in Figure 10. When the cell is at 40 °C, the linear absorption contrasts of three optical length structures are all small and their differences are negligible. As the cell temperature increases, the three curves all rise dramatically, while the linear absorption contrast of *l* = 10 mm cell maintains the highest and the through cell (*l* = 0.9 mm) has the lowest linear absorption contrast. Despite the glass reflection loss, rubidium condensation, and other factors, the relationships among the linear absorption contrasts of three configurations are not strictly linearly proportional to the optical length. At ~50 °C, the linear absorption contrast of *l* = 10 mm cell is nearly 10 times as large as that of the through cell (*l* = 0.9 mm). This 10-times relationship is not always established among the temperature range due to the contrast maximum limit, the nonlinear relationship of atomic density with temperature, etc. The operating temperature in chip scale atomic sensors for the conventional through cell architecture is ~90 °C. At that temperature, it is already completely optically thick for the return reflection architectures to achieve a linear absorption contrast improvement of 50% over that of the through cell architecture. The results also indicate the potential of reducing the operating temperature by 20–30 °C in chip scale atomic sensors to realize the similar linear absorption contrast, which results in power consumption reduction and the better performance of anti-relaxation wall coatings.

### 4.6. Through Cell CPT Measurement

**Figure 11:**
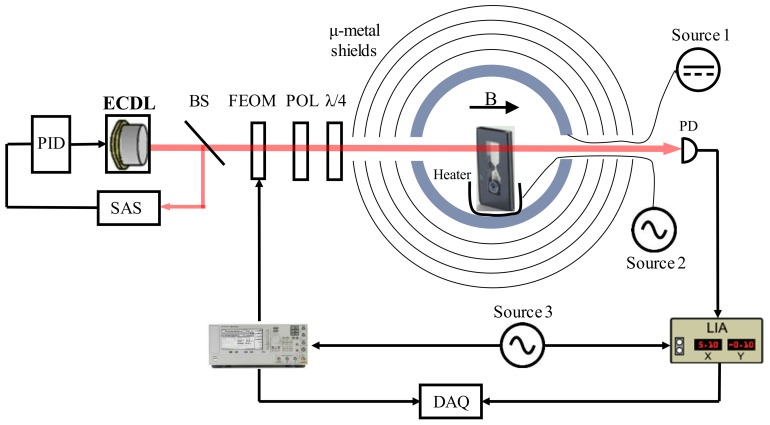
Schematic of the coherent population trapping (CPT) measurement apparatus. External cavity diode laser (ECDL): DLCpro, Toptica (Farmington, NY, USA). BS: Beam splitter. POL: Linear polarizer. PD: Photodiode. Source 1: Precision current driver. Source 2: Voltage supplier. Source 3: Function generator. Microwave generator: Agilent E8257D PSG analog signal generator (Santa Clara, CA, USA). LIA: LI5640 lock-in amplifier (NF Corporation, Yokohama, Japan).

To characterize the microfabricated vapor cells with more sensitive measurements, CPT spectroscopy experiments in the through cell optical configuration were implemented. The schematic of the apparatus is depicted in Figure 11. A 795 nm external cavity diode laser (ECDL) is used as the light source. It produces ~23.66 μW light in a 5 mm beam diameter. The laser is tuned to Rubidium-87 D1 line F = 1→F’ transition and saturation absorption spectroscopy (SAS) is applied for laser frequency stabilization. To establish the three-energy level structure and to prepare two coherent lights, the beam is coupled into the optical fiber and modulated by a Fiber Electro-Optic Modulator (FEOM), which is driven at 6.835 GHz by a microwave generator. The light then travels through polarization optics (a polarizer and a quarter-wave plate for circular polarization) towards the vapor cell. After exiting the cell, the transmission spectroscopy is measured by a photodiode and the resonance error signal is acquired with a lock-in amplifier (LIA). A function generator provides the reference signal for the microwave generator and LIA. The signal from microwave generator and LIA is acquired by a data acquisition board (DAQ). 

**Figure 12:**
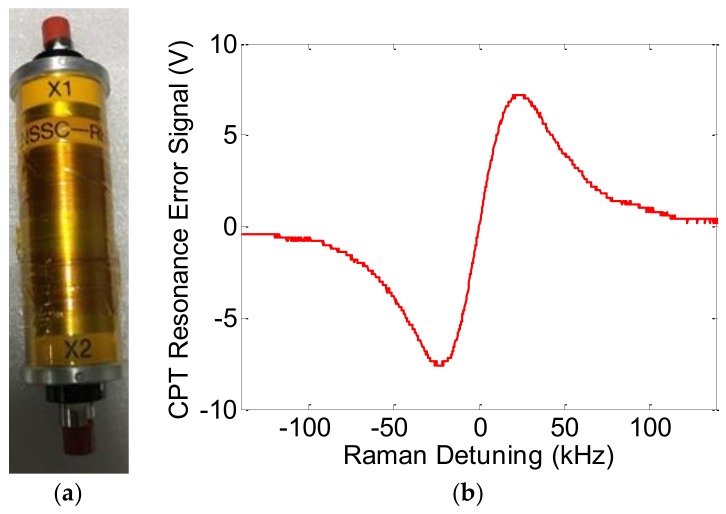
(**a**) Photo of the integrated physics package with polarization optics and the vapor cell; (**b**) Typical CPT 0-0 resonance error signal.

The microfabricated vapor cell with 5 mm reflective optical length (*l* = 5 mm) was placed at a custom bench in the center of the four-layer μ-metal magnetic shields. Solenoid coils are designed inside the innermost layer in order to provide a static magnetic field that is set to ~50 μT parallel to the direction of light propagation. The cell was heated to ~74 °C by a custom thermostatic heating unit to increase the rubidium atomic density, while the vapor is far from optically thick. The vapor cell and polarization optics are integrated in a custom physics package (all-optical sensor head) with fiber-coupled input and output light. Figure 12a shows the integrated physics package that is designed for glass-blown vapor cells. Here, we replace the conventional large cells with microfabricated vapor cells, which has the potential to minimize the volume of integrated physics package a lot. The typical 0-0 CPT resonance error signal obtained from LIA is presented in Figure 12b and the measured CPT signal linewidth (Full Width at Half Maximum, FWHM) is 46.3 kHz. Note that these microfabricated vapor cells can be filled with buffer gas to reduce the spin relaxation rate and narrow the linewidth down to ~10 kHz [62]. 

**Figure 13:**
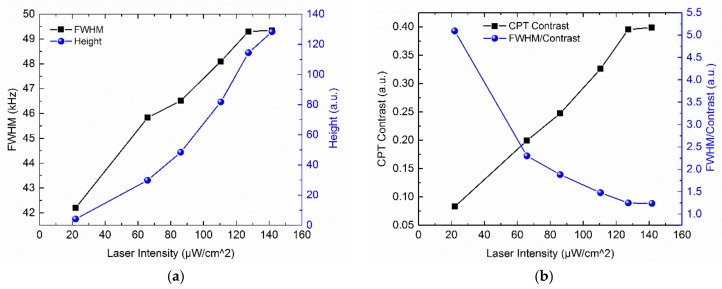
Measured (**a**) Full Width at Half Maximum (FWHM), height; (**b**) CPT signal contrast and FWHM/Contrast of the (0,0) CPT signal as a function of laser intensity for the microfabricated vapor cell.

The CPT resonance peak is in Lorentzian line-shape. Its *FWHM* and height *H* are given by [63]:

(7) $$FWHM\;=\;\frac{1}{\pi }\left(\gamma_{2}+\frac{\omega_{R}^{2}}{\Gamma^{*}}\right)$$

(8) $$H\propto n_{Rb}\frac{\omega_{R}^{4}}{\Gamma^{*}\left(\gamma_{2}+\frac{\omega_{R}^{2}}{\Gamma^{*}}\right)}$$

where *γ*_2_ is the transverse relaxation rate which is mainly the wall collision rate in our case, Γ* is the decay rate of the excited state, *ω_R_* is the Rabi frequency where its square is proportional to the laser intensity as $\omega_{R}^{2}\propto \;I$, and *n_Rb_* represents rubidium atomic density. The measured FWHM and height of the (0,0) CPT signal are presented in Figure 13a. For low intensities, FWHM increases linearly with laser intensity due to power broadening and the fit function of FWHM-Intensity dependence is (41.3 + 0.061 × *I*) kHz, which corresponds to the null power linewidth of 41.3 kHz. The CPT resonance height increases greatly with laser intensity in the low intensity regime and the fit function of Height-Intensity dependence is (*H* = 1.3376 + 0.0066 × *I*^2^). 

Due to the poor signal-to-noise ratio in time domain, it is more convenient for comparison and analysis to use CPT contrast defined as the height of CPT signal, divided by background signal far detuning from resonance. In the experiment, the background signal is estimated by the transmitted background optical power. For the chip scale atomic clock, the short-term frequency stability *σ_y_*(*τ*) can be given by [64]:

(9) $$\sigma_{y}\left(\tau \right)\propto \frac{FWHM}{CPT\;Contrast}\tau^{-1/2}$$

where *τ* is the integration time. Equation (9) means improving the chip scale atomic clock performance requires minimizing FWHM/Contrast. The measured (0,0) CPT resonance contrast and FWHM/Contrast are presented in Figure 13b. For low intensities, the height of CPT signal increases faster than background signal with laser intensity, leading to the increase of CPT contrast. When the intensity is closer to saturation, the CPT contrast increases slower. In the low intensity regime, the value of FWHM/Contrast decreases with laser intensity and its reduction rate gradually becomes smaller in order to minimize FWHM/Contrast as well as the short-term frequency stability. The CPT measurement proves the effectiveness of the microfabricated vapor cell in through cell optical configuration.

## 5. Conclusions

In summary, we have investigated the miniature vapor cells with reflective sidewalls for the applications of chip scale atomic sensors. The light is redirected twice by the aluminum reflectors on the wet etched 54.7° sidewalls and returns to the emission plane. Based on MEMS technology and integrated optics, the proposed vapor cell is suitable for single-beam applications, such as atomic clocks, as well as pump-and-probe-beam applications, such as atomic magnetometers. The elongated vapor cell is compliant with the MEMS fabrication assembly and an appropriate bulk silicon microfabrication process as well as alkali metal encapsulation has been demonstrated. To avoid limitations that are faced in the fabrication process, a simpler, more universal, and less constrained fabrication process of chip scale atomic sensors with uncompromised performance is implemented, which also decreases the fabrication costs and procedures. In order to demonstrate the reduction of fabrication complexity, different tests have been carried out. The miniaturized vapor cells had a volume of ~50 mm^3^ and a leak rate of 1.5 × 10^−8^ Pa·m^3^/s leading to an acceptable lifetime. The aluminum sidewall reflectance was measured to be twice as much as that of bare silicon. With two paired aluminum reflectors to reflect light twice, the total reflectance was nearly three times that of uncoated silicon reflectors and the state of polarization was preserved. The through cell with 0.9 mm optical length as well as elongated cells with 5 mm and 10 mm optical lengths were measured by absorption spectroscopy experiments of rubidium D1 line. The strengths of atomic transitions were characterized. The linear absorption contrasts of through cell, 5 mm and 10 mm optical lengths were compared to evaluate the efficiency due to sidewall reflection. Under conditions of effective sidewall reflectors, the linear absorption contrast has been improved by as much as 10 times by integrating the aluminum reflectors at ~50 °C. A 50% linear absorption contrast increase over the tradition MEMS vapor cell at the operating temperature ~90 °C was obtained. This indicates the potential of more competitive clock stability and magnetometer sensitivity, as well as lower power consumption in chip scale atomic sensors. Furthermore, the vacuum microfabricated cells were characterized by more sensitive CPT spectroscopy measurements. In the through cell configuration, a linewidth of 46.3 kHz and null-power linewidth down to 41.3 kHz were obtained due to the absence of buffer gas. The variation tendency of CPT signal height, CPT contrast, and FWHM/Contrast with laser intensity was investigated and consistent with theoretical analysis. This proves the effectiveness of vacuum microfabricated cells in chip scale atomic sensors.
